# Multi-cue temporal modeling for skeleton-based sign language recognition

**DOI:** 10.3389/fnins.2023.1148191

**Published:** 2023-04-05

**Authors:** Oğulcan Özdemir, İnci M. Baytaş, Lale Akarun

**Affiliations:** Perceptual Intelligence Laboratory, Computer Engineering Department, Boğaziçi University, Istanbul, Türkiye

**Keywords:** sign language recognition, spatio-temporal representation learning, graph convolutional networks, long short-term memory networks, deep learning-based human action recognition

## Abstract

Sign languages are visual languages used as the primary communication medium for the Deaf community. The signs comprise manual and non-manual articulators such as hand shapes, upper body movement, and facial expressions. Sign Language Recognition (SLR) aims to learn spatial and temporal representations from the videos of the signs. Most SLR studies focus on manual features often extracted from the shape of the dominant hand or the entire frame. However, facial expressions combined with hand and body gestures may also play a significant role in discriminating the context represented in the sign videos. In this study, we propose an isolated SLR framework based on Spatial-Temporal Graph Convolutional Networks (ST-GCNs) and Multi-Cue Long Short-Term Memorys (MC-LSTMs) to exploit multi-articulatory (e.g., body, hands, and face) information for recognizing sign glosses. We train an ST-GCN model for learning representations from the upper body and hands. Meanwhile, spatial embeddings of hand shape and facial expression cues are extracted from Convolutional Neural Networks (CNNs) pre-trained on large-scale hand and facial expression datasets. Thus, the proposed framework coupling ST-GCNs with MC-LSTMs for multi-articulatory temporal modeling can provide insights into the contribution of each visual Sign Language (SL) cue to recognition performance. To evaluate the proposed framework, we conducted extensive analyzes on two Turkish SL benchmark datasets with different linguistic properties, BosphorusSign22k and AUTSL. While we obtained comparable recognition performance with the skeleton-based state-of-the-art, we observe that incorporating multiple visual SL cues improves the recognition performance, especially in certain sign classes where multi-cue information is vital. The code is available at: https://github.com/ogulcanozdemir/multicue-slr.

## 1. Introduction

Sign Languages (SLs) are multi-cue visual languages that have naturally emerged as the primary communication medium among the Deaf. The multi-cue nature stems from manual (e.g., hand gestures, hand shapes) and non-manual features (e.g., facial expressions, mouthing, mouth gestures, upper body movements). This visual communication is a proper, full-fledged language possessing all linguistic components (Stokoe, 2005; Sandler and Lillo-Martin, 2006). The linguistics of this language, as well as its production and comprehension in the human brain, has attracted intense research interest (Campbell et al., 2008; Emmorey, 2021). It has been shown that the human brain processes SLs using similar brain organization patterns as it does for spoken languages (Campbell et al., 2008). Nevertheless, differences in the perception of certain aspects, such as non-manual components and iconic signs, as well as in the use of brain areas between the Deaf and hearing native signers, continue to be active research areas (Emmorey, 2021).

Although SLs enable the Deaf to communicate, there is a prevalent disconnection between deaf signers and people who cannot sign. It is crucial to improve the accessibility to SL communication and education resources to facilitate inclusiveness for the Deaf. On the other hand, the number of SL interpreters and tutors is insufficient. To that end, Automatic Sign Language Recognition (ASLR) systems have been designed to mitigate such challenges in SL education and communication. The ASLR by computers has been the subject of research efforts for almost 30 years (Loeding et al., 2004).

The ASLR is an integral component of sign language translation and animation frameworks. Thus, it also facilitates the creation of digital content for deaf communities (Ferreira et al., 2021). These systems often focus on recognizing and translating the multiple visual cues of a signer performing in front of a camera. For this reason, the ASLR task can be posed as a spatio-temporal representation learning problem. Over the last 30 years, researchers have worked on different sub-tasks of ASLR. Recognizing isolated SL glosses, where a single word is associated with the sign, has been a matter of interest since the earlier studies in the SLR field (Liwicki and Everingham, 2009; Kındıroğlu et al., 2019). Additionally, employing models that recognize and translate continuous SL videos, where more than one sign gloss is present, and producing SL gloss sentences from spoken language sentences have become popular in recent studies (Camgoz et al., 2017, 2018, 2020b; Pu et al., 2019; Saunders et al., 2021).

In this study, we focus on the isolated SLR task where a single sign gloss, i.e., a word associated with the sign, is performed in the input video. Despite its similarity to the human action recognition problem, SLR focuses more on local cues of hand gestures and shapes, facial expressions, mouth gestures, and mouthing. Therefore, exploiting the multiple cues in spatio-temproral architectures may improve the ASLR performance. To thoroughly leverage their contributions, we need distinctive representations of the cues. While earlier studies utilized handcrafted feature extraction techniques for training and inference (Peng et al., 2015), the availability of isolated SL datasets has enabled researchers to develop deep learning-based ASLR approaches (Zhang et al., 2016; Joze and Koller, 2018; Albanie et al., 2020; Özdemir et al., 2020; Sincan and Keles, 2020). Inspired by the architectures on the human action recognition domain, researchers have also investigated employing spatio-temporal approaches that use 2D and 3D CNNs and Long Short-Term Memorys (LSTMs) for the ASLR task (Huang et al., 2015; Koller et al., 2016; Liu et al., 2016; Joze and Koller, 2018; Boháček and Hrúz, 2022; Hrúz et al., 2022).

**Figure 1:**
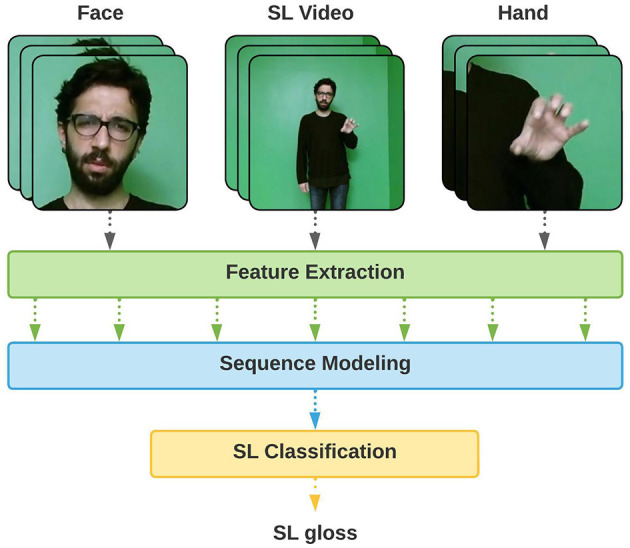
Multi-cue isolated sign language recognition (SLR).

These architectures often operate in an end-to-end manner, where the model can learn all steps jointly, to recognize sign glosses by utilizing manual features such as the shape of the dominant hand or the entire frame (Joze and Koller, 2018; Özdemir et al., 2020). However, SLs are considered multi-cue languages, where each channel contains a manual or non-manual characteristic. For this reason, utilizing a single visual cue, such as only manual features, may not be sufficient to fully express the context of the SL videos. In addition to the improved recognition performance when facial expressions, mouthing, and upper body movements are incorporated with a multi-cue recognition architecture, considering multiple cues also facilitates identifying the individual contributions of manual and non-manual features to the recognition task (Figure 1).

More recently, the success of pose estimation techniques and Graph Convolutional Network (GCN) architectures has shifted researchers' attention to skeleton-based approaches in both action recognition and SLR domains (Kipf and Welling, 2016; Yan et al., 2018; Cao et al., 2019; Jiang et al., 2021). In these methods, graphs are often formed by connecting skeleton joint information (obtained *via* pose estimation techniques) according to the natural human body connections and processed through a GCN network. As an improvement over earlier GCN architectures, ST-GCN has been proposed for skeleton-based action recognition to model spatial and temporal dimensions simultaneously and later was adapted to the SLR problem (Yan et al., 2018; Jiang et al., 2021).

In this paper, we propose a multi-cue SLR architecture utilizing multi-articulatory spatio-temporal information contained in manual and non-manual features of SL, as illustrated in Figure 1. The proposed architecture comprises a spatio-temporal feature extraction module, which aims to extract visual representations of the multiple cues using ST-GCNs and pre-trained CNNs and an LSTM-based temporal modeling module that jointly learns temporal interactions between the multiple cues.

- A new multi-cue sequential architecture, MC-LSTM, is designed as an extension of Multi-View Long Short-Term Memorys (MV-LSTMs). MC-LSTM introduces an adaptive fusion mechanism to learn how to fuse the information transferred *via* the representations of the multiple SL cues. In particular, multiple cues are integrated within the temporal representation module to model the asynchronous interactions between each cue during training.
- An ST-GCN architecture is trained jointly with MC-LSTM to learn spatio-temporal information from full-body skeleton joints, and features obtained from facial and hand representations using pre-trained CNNs.
- To the best of our knowledge, this is the first attempt to model manual and non-manual cues in SLs with hybrid architectures containing ST-GCN and LSTMs. Furthermore, the adaptive fusion mechanism introduced in MC-LSTM learns how much information should be included from each cue at each time step.
- An extensive ablation study is presented for different modules of the proposed SL recognition framework, where the contributions of different visual SL cues to recognition performance are investigated.
- An analysis is performed on different sign language-specific linguistic attributes to examine the benefits of utilizing the proposed multi-cue sequential architecture for the SLR task.

We evaluate our methods on two Turkish SL benchmark datasets, BosphorusSign22k (Özdemir et al., 2020) and AUTSL (Sincan and Keles, 2020), that present different characteristics and challenges. Our ablation study demonstrates that incorporating multiple visual SL cues improves recognition performance.

The rest of this paper is organized as follows; we briefly review the SLR, skeleton-based representation learning, and multi-channel sequence modeling literature in Section 2. In section 3, we introduce our multi-cue spatio-temporal architecture for recognizing SLs. We then describe our implementation and share quantitative and qualitative results on both BosphorusSign22k and AUTSL, isolated SLR benchmark datasets, in Section 4. Finally, we discuss our results and conclude the paper in Section 5.

## 2. Related work

In this section, we present the prominent studies in isolated SLR. Then, we continue with a detailed review of skeleton-based representation learning and temporal modeling studies.

### 2.1. Sign language recognition

Sign Language Recognition (SLR) has been studied in the pattern recognition domain for over 30 years since it is imperative to alleviate the communication barrier between deaf and hearing communities (Loeding et al., 2004). In earlier studies, researchers often focused on recognizing hand gestures using the information obtained through hand sensors (Kadous, 1996; Vogler and Metaxas, 1997; Hienz et al., 1999; Hernandez-Rebollar et al., 2002). Following the increased availability of vision-based and depth cameras, researchers started to design more sophisticated approaches that facilitate learning information from videos. These approaches aimed to learn representations related to SLs, such as hand shapes, motion, and upper body information (Liu and Fujimura, 2004; Wong and Cipolla, 2005; Liwicki and Everingham, 2009; Shotton et al., 2011; Nandakumar et al., 2013; Camgöz et al., 2016a; Koller et al., 2016; Özdemir et al., 2016, 2018).

Earlier research on SLR has mostly concentrated on approaches in the isolated SLR domain, where sign videos of signers typically facing the camera and performing a sign gloss are recognized. Researchers have first employed handcrafted methods to extract representations describing localized regions of manual features (hand shapes) or the information from the entire frame (Nandakumar et al., 2013; Peng et al., 2015; Camgöz et al., 2016a; Özdemir et al., 2018). Since it is a video classification task, earlier work on SLR has adapted well-known human action recognition techniques to the domain. Özdemir et al. (2016) and Peng et al. (2015) used Improved Dense Trajectory (IDT) handcrafted features to recognize isolated sign videos. Due to the high computational complexity of IDT for the SLR task, Özdemir et al. (2018) later proposed an efficient approach by obtaining similar sign language descriptors from localized regions of hand shapes.

More recently, SLR researchers have shifted their attention to the successful spatio-temporal deep learning approaches in which they employ 2D and 3D CNNs (Huang et al., 2015; Camgöz et al., 2016b; Koller et al., 2016; Joze and Koller, 2018; Mittal et al., 2019; Gökçe et al., 2020; Rastgoo et al., 2020; Abdullahi and Chamnongthai, 2022; Samaan et al., 2022). Moreover, Kındıroğlu et al. (2019), Kındıroglu et al. (2023) have proposed a temporal representation approach for modeling the movement-hold pattern of SLs (Liddell and Johnson, 1989), in which authors adapted the pose-based motion representation, PoTion (Choutas et al., 2018), to the SLR task.

### 2.2. Skeleton-based representation learning

Although the architectures based on CNNs and Recurrent Neural Networks (RNNs) were successful in earlier studies of video classification tasks such as SLR and human action recognition, utilizing skeleton data obtained from pose estimation techniques has become more popular recently (Chéron et al., 2015; Du et al., 2015; Lev et al., 2016; Li et al., 2017; Liu et al., 2017; Wang and Wang, 2017; Liang et al., 2018; Zhu et al., 2019). Compared to frame-based inputs, the skeleton information has more representational power to express the localized motion centered around the sign performed. Since the skeleton data information can be more robust to changes in the environment and can be represented more compactly than frame-based representations, researchers have moved their interest into developing skeleton-based approaches (Song et al., 2022).

The earlier work has often focused on learning skeleton-based information from their meaningful visual representations. However, the proposed architectures can not be generalized to the spatio-temporal skeleton joint information without excessive pre-preprocessing (Kındıroğlu et al., 2019). As several approaches have been proposed to model spatio-temporal graph-structured data for the traffic flow prediction problem in the literature (Li et al., 2019; Hou et al., 2021; Wu et al., 2021), proposed methods employ mixed GCN and LSTM architectures which are computationally inefficient for skeleton-based recognition tasks since approaches require modeling different topologies at each time step. To model the skeleton joint information, Yan et al. (2018) proposed ST-GCNs for learning both spatial and temporal dynamics of the human skeleton for the skeleton-based human action recognition task. In their work, authors adapted GCNs to operate and learn on graph-structured information for the spatio-temporal representation domain. After the success of ST-GCNs on the temporal modeling tasks, improvements have also been proposed to solve several drawbacks of ST-GCNs, such as occlusions and modeling the distant joint relationships (Wang et al., 2018; Si et al., 2019; Song et al., 2019, 2020; Zhang et al., 2019; Liu et al., 2020; Plizzari et al., 2021; Lee et al., 2022).

ST-GCN architecture has also been adapted into the SLR domain (de Amorim et al., 2019; Jiang et al., 2021; Tunga et al., 2021; Vazquez-Enriquez et al., 2021). de Amorim et al. (2019) have proposed adapting baseline ST-GCNs architecture into the SLR domain on a small subset of the ASLLVD-Skeleton dataset (Neidle et al., 2012). Jiang et al. (2021) followed a multi-modal ensemble approach in which they coupled the skeleton information with RGB frame, optical flow, and depth information. Moreover, Vazquez-Enriquez et al. (2021) employed a multi-scale variant of ST-GCNs on the AUTSL dataset. They investigated the effects of transfer learning on the isolated SLR task by focusing on training their architecture on AUTSL, WLASL, and LSE_UVIGO datasets (Doćıo-Fernández et al., 2020; Li et al., 2020; Sincan and Keles, 2020). More recently, Tunga et al. (2021) proposed a mixed GCN and Bidirectional Encoder Representations from Transformers (BERT) (Devlin et al., 2018) architecture to capture pose-based skeleton information and model temporal dependencies between each time step.

### 2.3. Temporal modeling

Due to its sequential nature, the SLR task requires temporal modeling to learn the structures of sign glosses varying over time. While the prior SL literature focuses more on techniques such as Hidden Markov Models (HMMs) for sequence modeling after extracting handcrafted features, recent studies follow the idea of employing 2D-3D CNN and RNN-based architectures in which frames or skeleton joint information are directly used (Aran, 2008; Camgöz et al., 2016a; Koller et al., 2016, 2019; Zhang et al., 2016; Mittal et al., 2019; Abdullahi and Chamnongthai, 2022; Samaan et al., 2022). More recently, Transformer based architectures have become popular on SLR and Sign Language Translation (SLT) tasks due to their success in domains such as Natural Language Processing (NLP) and Speech Processing (SP) (Vaswani et al., 2017; Camgoz et al., 2020b; Rastgoo et al., 2020; Boháček and Hrúz, 2022; Cao et al., 2022; Chen et al., 2022; Hrúz et al., 2022; Hu et al., 2022; Xie et al., 2023).

Although LSTM and 3D-CNN-based sequential architectures can learn strong spatio-temporal representations from sign videos, they do not fully exploit the multi-articulatory nature of SLs using only manual features such as hand shape or gestures. While the LSTM is often used as a sequential modeling method (Hochreiter and Schmidhuber, 1997), it cannot inherently process multiple channels of information without performing early or late fusion. Rajagopalan et al. (2016) have proposed MV-LSTMs as an extension for LSTMs for multi-view sequences. In their work, they modified LSTM cells to learn the interactions between multiple channels by partitioning the memory cell using predetermined view interaction terms. Similarly, Camgoz et al. (2020a) employed multi-channel transformers for the SLT task, where the architecture learns from multiple channels using a modified Transformer architecture (Chang et al., 2021). Recently, Li and Meng (2022) proposed a Transformer-based multi-channel architecture using the information from the entire frame and skeleton input data for the SLT task.

In this paper, we aim to employ a hybrid ST-GCN and MC-LSTM architecture which introduces an adaptive fusion of multiple cues in SLs learned from data during training. Since MC-LSTMs are adopted as an extension to MV-LSTM, a direct contribution of each visual SL cue to the sequence modeling is facilitated. Our analysis compares our hybrid architecture with ST-GCN and LSTM baselines on isolated SLR datasets, BosphorusSign22k and AUTSL.

## 3. Method

**Figure 2:**
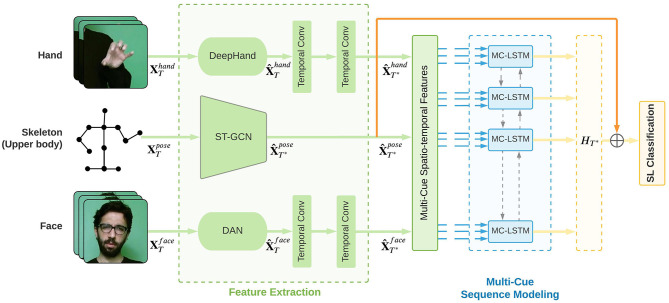
Overview of our proposed multi-cue SLR architecture. ST-GCN and temporal convolutional layers are trained jointly with MC-LSTM, where cue-specific and cross-cue parameters are learned.

The proposed architecture in Figure 2 comprises spatial and temporal representation learning modules. In particular, an ST-GCN module is designed to extract spatio-temporal features from the full-frame cue using pose estimation information, pre-trained CNN modules are used to extract spatial representations from the hand and the face cue, and an MC-LSTM temporal modeling module is proposed to learn how to fuse multiple temporal visual cues for the isolated SLR task. Finally, the learned spatio-temporal representation is mapped to a sign gloss. In the following section, each module is presented in detail.

### 3.1. Spatio-temporal feature extraction

To obtain powerful representations, we utilize ST-GCN for skeleton joint features obtained from an open-source pose estimation toolbox and extract features from pre-trained CNN architectures suitable for the corresponding visual SL cues. Let a given visual cue sequence of length *T* be expressed as ${\text{X}}_{T}^{cue}=\left\{{\text{x}}_{1}^{cue},{\text{x}}_{2}^{cue},{\text{x}}_{3}^{cue},...,{\text{x}}_{T}^{cue}\right\}$. The pose cue is the upper body pose information ${\text{X}}_{T}^{pose}\in {\mathbb{R}}^{T\times j\times 3}$, where *j* is the number of unique joints, and the hand and face cues are the hand and face crop images obtained from SL frames, ${\text{X}}_{T}^{hand,face}\in {\mathbb{R}}^{T\times h\times w\times 3}$, where *h* × *w* is the crop resolution of RGB images. We pass all inputs through a feature extraction module:


(1) $$\begin{array}{l} {\hat{x}}_{t}^{cue}=F^{cue}\left({\text{x}}_{t}^{cue}\right)\;t=1,2,3,...,T^{*} \end{array}$$

(2) $$\begin{array}{l} {\hat{X}}_{T^{*}}^{cue}=\left[{\hat{x}}_{1}^{cue},{\hat{x}}_{2}^{cue},...,{\hat{x}}_{T^{*}}^{cue}\right] \end{array}$$

Where *F*^*cue*^ is the architecture selected for the visual cue, and ${\hat{x}}_{t}^{cue}$ is the output feature of the visual cue at time *t*. When the upper body pose is concerned, *F*^*cue*^ denotes the ST-GCN, which is trained from scratch simultaneously with the temporal module. On the other hand, for hand shape and facial expression features, *F*^*cue*^ denotes the pre-trained architectures. Since ST-GCNs temporally pool information on its intermediate layers, we also perform temporal pooling over all features obtained from other visual cues (e.g., hand and face cues) to adjust the length of the features from *T* to T^*^ (T^*^ < *T*), where T^*^ indicates the length of the visual cue features after the temporal pooling. Once we obtain all the features for multiple visual cues, we form a feature sequence ${\hat{X}}^{cue}$ for each cue with the dimensions of ${\hat{X}}_{T^{*}}^{pose}\in {\mathbb{R}}^{T\times p}$ where *p* = 256 is the output size of the ST-GCN, and ${\hat{X}}_{T^{*}}^{hand,face}\in {\mathbb{R}}^{T\times d}$ where *d* ∈ {512, 1024} is the output size of hand and face representation architectures, respectively. Then, the feature sequences will be fed into the proposed sequential MC-LSTM module.

#### Spatial-temporal graph convolutional networks (ST-GCNs)

ST-GCN architecture (Yan et al., 2018), an extension of GCNs (Kipf and Welling, 2016) to both spatial and temporal dimensions, comprises stacked spatio-temporal blocks that process skeleton graphs using spatial graph convolutions (GCN) and temporal convolutions (TCN).

Skeleton joints contain vital information regarding the spatial and temporal dynamics of the body movement constituting the sign. ST-GCNs are shown to capture such dynamics better than an RGB-based architecture (Yan et al., 2018; Song et al., 2022). For this reason, we integrate an ST-GCN module to model the upper body movements. Following Yan et al. (2018), we construct a spatio-temporal skeleton graph *G* = (*V, E*) using upper body and hand skeleton joints with 35 unique joints for the skeleton sequence of length *T*. While *V* is the set of nodes including the (*x*, *y*) coordinates and confidence values of all selected joints at each time step *t*, *E* is the edge set consisting of two subsets; the natural human body part connections and temporal trajectories connecting respective joints over time.

We propose several improvements to the baseline ST-GCN architecture, making it more versatile such that the temporal pooling of the ST-GCN module does not completely collapse the temporal dimension at the end. Thus, the spatio-temporal representation from the full frame learned with the proposed ST-GCN can be fed to the MC-LSTMs. In particular, we first adjust the temporal kernel size *t*_*k*_ of all blocks and the temporal stride *t*_*s*_ of the convolutional blocks where the number of channels in feature maps is increased (the ablation study for adjusting the parameters is provided in the Supplementary material). Secondly, as the baseline ST-GCN architecture collapses all temporal information before classification, we use the output representation ${\hat{X}}_{T^{*}}^{pose}$ of the last ST-GCN block before temporal average pooling, and feed this into our sequential module. With the ST-GCN architecture, we aim to capture spatio-temporal patterns composing the signing action invariant to the signer and the background.

#### CNN-based hand and facial expression representations

As discussed earlier, SLs have a multi-cue nature. The representation learned for the action in the entire frame may overlook the characteristic details retained in the manual and non-manual articulators, namely, hand shapes and facial expressions. To address this issue, we propose incorporating features extracted from localized regions of hand shapes and facial expressions into the recognition framework. On the other hand, learning to extract hand and face features simultaneously with learning spatio-temporal representations may not serve our purpose due to the limited data in the SLR domain. For this reason, we propose to extract hand and facial features from CNN architectures pre-trained exclusively for facial expression (Wen et al., 2021) and hand shape (Koller et al., 2016) recognition.

After extracting the features ${\hat{X}}_{T}^{cue}$ from task specific pre-trained architectures, we learn two temporal convolutional layers of kernel sizes *t*_*k*_ and strides *t*_*s*_ (same as in ST-GCN) for reducing the length of sequences to T^*^ for hand and facial expressions, identically to the output sequence length of the ST-GCN architecture. Then, we feed temporally pooled feature representations (e.g., ${\hat{X}}_{T^{*}}^{hand}$, ${\hat{X}}_{T^{*}}^{face}$) into our sequential model. For facial expression representation, we employ Distract your Attention Network (DAN) (Wen et al., 2021) architecture, pre-trained on the widely used AffectNet (Mollahosseini et al., 2017) dataset. For hand shape representations, we extract features from the DeepHand (Koller et al., 2016) architecture which is trained over one million hand images.

### 3.2. Multi-cue temporal modeling

In the SLR task, temporal dependencies carry essential linguistic information about the sign gloss. Although RNNs can learn complex temporal dynamics, in theory, they cannot capture long-term dependencies due to the vanishing gradients problem (Pascanu et al., 2013). For modeling the long-term dependencies, Hochreiter and Schmidhuber (1997) proposed LSTMs capable of learning long sequences by regulating the long-term and short-term memory with several gates.

Given an input feature sequence $\hat{X}=\left\{{\hat{x}}_{1},{\hat{x}}_{2},...,{\hat{x}}_{T}\right\}$ of length *T*, LSTM cell updates at time *t* are calculated using the previous hidden state *h*_*t*−1_ and cell state *c*_*t*−1_ as shown below:


(3) $$\begin{array}{l} i_{t}=\sigma \left(W_{ix}{\hat{x}}_{t}+W_{ih}h_{t-1}+b_{i}\right) \end{array}$$

(4) $$\begin{array}{l} f_{t}=\sigma \left(W_{fx}{\hat{x}}_{t}+W_{fh}h_{t-1}+b_{f}\right) \end{array}$$

(5) $$\begin{array}{l} o_{t}=\sigma \left(W_{ox}{\hat{x}}_{t}+W_{oh}h_{t-1}+b_{o}\right) \end{array}$$

(6) $$\begin{array}{l} \tilde{c_{t}}=\text{tanh}\left(W_{ci}{\hat{x}}_{t}+W_{ch}h_{t-1}+b_{c}\right) \end{array}$$

(7) $$\begin{array}{l} c_{t}=f_{t}⊙c_{t-1}+i_{t}⊙\tilde{c_{t}} \end{array}$$

(8) $$\begin{array}{l} h_{t}=o_{t}⊙\text{tanh}\left(c_{t}\right) \end{array}$$

Where *i*_*t*_, *f*_*t*_ and *o*_*t*_ are input, forget and output gates, respectively, $\tilde{c_{t}}$ denotes the cell state update, and *h*_*t*_ and *c*_*t*_ are the current time step's hidden and cell states. LSTMs are inherently incompatible with inputs containing multiple channels (or modalities).

To introduce multi-view property, Rajagopalan et al. (2016) proposed MV-LSTMs, a modified LSTM model that partitions the memory into view-specific ***V***_***s***_ and cross-view ***V***_***c***_ components. In their work, authors aimed to partition cell states and form flexible interaction between multiple channels of information to overcome the adverse effects of dominating modalities. In order to achieve this, they considered using pre-defined view-specific (***V***_***s***_) and cross-view (***V***_***c***_) interaction terms split accordingly to preset α and β scalar matrices (Rajagopalan et al., 2016).

In this study, we introduce MC-LSTMs by extending MV-LSTMs into our isolated SLR framework to model the information conveyed through multiple visual cues explicitly; upper body skeleton, hand shape, and facial expressions.

Considering the nature of SLs, it is not possible to know which visual cue will carry the essential information for the corresponding time step. Hand shape might be the most informative cue for a particular time window, while facial expression or body gesture might carry more distinctive information for another time window. For this reason, pre-determined ***V***_***s***_ and ***V***_***c***_ are not suitable for SLR. To address this challenge, we adjust MV-LSTMs (Rajagopalan et al., 2016) cell structure with trainable ***V***_***s***_ (cue-specific) and ***V***_***c***_ (cross-cue) parameters to learn the interaction mapping between the different visual cues during training.

For a given input SL feature sequence $\hat{X}={\left\{{\hat{x}}_{1},{\hat{x}}_{2},...,{\hat{x}}_{T^{*}}\right\}}_{cue=1}^{N}$ of length T^*^ (see Equation 2) with *N* visual cues, a single-cue cell update at time *t* for a visual cue is defined as the following:


(9) $$i_{t}^{cue}=\sigma (W_{ix}^{cue}{\hat{x}}_{t}^{cue}+W_{ih}^{cue}V_{s}h_{t-1}^{cue}+\sum_{j=1,j\neq cue}^{N}W_{ih}^{j}V_{c}h_{t-1}^{j})$$

(10) $$f_{t}^{cue}=\sigma (W_{fx}^{cue}{\hat{x}}_{t}^{cue}+W_{fh}^{cue}V_{s}h_{t-1}^{cue}+\sum_{j=1,j\neq cue}^{N}W_{fh}^{j}V_{c}h_{t-1}^{j})$$

(11) $$o_{t}^{cue}=\sigma (W_{ox}^{cue}{\hat{x}}_{t}^{cue}+W_{oh}^{cue}V_{s}h_{t-1}^{cue}+\sum_{j=1,j\neq cue}^{N}W_{oh}^{j}V_{c}h_{t-1}^{j})$$

(12) $${\tilde{c}}_{t}^{cue}=\text{tanh}(W_{cx}^{cue}{\hat{x}}_{t}^{cue}+W_{ch}^{cue}V_{s}h_{t-1}^{cue}+\sum_{j=1,j\neq cue}^{N}W_{ch}^{j}V_{c}h_{t-1}^{j})$$

(13) $$\begin{array}{l} c_{t}^{cue}=f_{t}^{cue}⊙c_{t-1}^{cue}+i_{t}^{cue}⊙{\tilde{c}}_{t}^{cue} \end{array}$$

(14) $$\begin{array}{l} h_{t}^{cue}=o_{t}^{cue}⊙\text{tanh}\left(c_{t}^{cue}\right)\;\text{where }cue\in \left\{\text{pose},\text{hand},\text{face}\right\} \end{array}$$

Where $i_{t}^{cue}$, $f_{t}^{cue}$ and $o_{t}^{cue}$ are input, forget and output gates of the cell belonging to a visual SL cue, and $h_{t-1}^{cue}$ and $c_{t-1}^{cue}$ are the hidden and cell states of the previous time step *t*−1 for the visual cue. Once MC-LSTM cell updates are performed for all the views, hidden states are then concatenated for all time steps:


(15) $$\begin{array}{l} H_{t}=\left[h_{t}^{1};h_{t}^{2};h_{t}^{3};...;h_{t}^{N}\right] \end{array}$$

(16) $$\begin{array}{l} H_{t}\in {\mathbb{R}}^{T^{*}\times N\times k\times 2} \end{array}$$

Where *k* × 2 is the output size of the bidirectional MC-LSTM architecture, *N* enumerates the different cues. As a final step, we average all output hidden states *H*_*t*_ over the entire output sequence with the length of T^*^, and perform multi-cue SL classification, where we optimize cross-entropy loss:


(17) $$\begin{array}{l} L_{ce}=-\overset{C}{\underset{c=1}{\sum }}y_{c}\log{\hat{y}}_{c} \end{array}$$

Where *C* is the number of sign classes, *y*_*c*_ and ŷ_*c*_ are the one-hot encoded ground truth vector and the prediction probabilities, respectively.

## 4. Experimental results

This section describes the datasets used to evaluate our architecture and experimental design, and presents our quantitative and qualitative results.

### 4.1. Datasets

**Figure 3:**
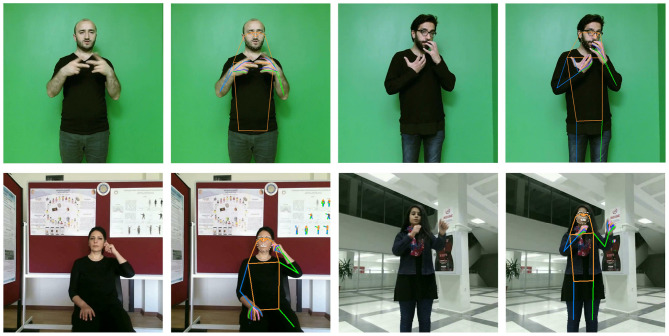
Example frames from SL videos and pose estimation outputs from the BosphorusSign22k **(top)** and AUTSL **(bottom)** datasets.

To evaluate our architecture, we used BosphorusSign22k (Özdemir et al., 2020) and AUTSL (Sincan and Keles, 2020) datasets in our experiments (examples are shown in Figure 3).

#### BosphorusSign22k

The dataset was recently published for isolated SLR and is publicly available upon request. It contains 22,542 trimmed videos of 744 SL gloss classes performed by six native signers repetitively in front of a camera and Chroma-Key background. SL videos in this dataset were captured at a high resolution of 1,920 × 1,080 at 30 frames per second. To make our analysis comparable to other studies on this dataset, we followed the training protocol in Özdemir et al. (2020) in which a single signer is selected for testing purposes while the rest of the signers are used for training and report Top-1 and Top-5 classification accuracies.

#### AUTSL

Introduced by Sincan and Keles (2020), the AUTSL dataset consists of 38,336 trimmed RGB and depth sign language videos from 226 sign glosses of Turkish SL. Sign videos in the dataset were performed by 43 different signers sitting or standing in front of the camera during recording each video, which is captured at a resolution of 512 × 512 at 30 frames per second. Since this dataset was introduced as a part of the ChaLearn Looking at People challenge (Sincan et al., 2021), it contains sign videos with different backgrounds from indoor and outdoor environments to make the recognition problem more challenging. Similar to our experiments with the BosphorusSign22k dataset, we followed the training protocol in Sincan et al. (2021), in which the dataset is split into three subsets: train, development, and test. We report Top-1 and Top-5 classification accuracies from training and test subsets to evaluate our approach.

Both datasets have been pre-processed by their authors before distribution so that the dominant hand is always the same. Although both datasets have similar characteristics related to the isolated SLR task, they have several differences. Firstly, the AUTSL dataset has varying backgrounds as opposed to the Chroma-Key background in BosphorusSign22k dataset. This difference might cause the features extracted from AUTSL to be variant to the changing factors in the background. Consequently, we observe the effects of AUTSL-specific challenges in recognition performance. Secondly, signers in the BosphorusSign22k dataset are all native signers; thus, videos in the dataset often have mouthing and mouth gestures which are imperative to exploit multi-channel information. However, similar facial gestures and mouthing do not exist in the AUTSL videos since they are not native. Moreover, in some of the AUTSL videos, signers sit while performing the signs. However, the signers in the BosphorusSign22k dataset always perform the signs while standing in front of the camera. Since standing and sitting actions change the position and pose of the upper body, we want the stress that representations learned for the AUTSL dataset may inevitably carry such variations.

#### Sign language specific linguistic attributes

In addition to the gloss recognition performance, we investigate the effects of different cues on the recognition performance of SL-specific linguistic attributes. For this purpose, we group signs with similar grammatical attributes, such as one-handed, two-handed, circular, non-circular, repetitive, non-repetitive, mono-morphemic, and compound signs. Our analysis for both datasets in **Tables 5**, **6** unfolds insights into how the architectures incorporating different cues behave under the specific groups of sign glosses.

### 4.2. Implementation details

In this section, we provide a detailed explanation of spatio-temporal feature extraction and training hyperparameters for the proposed isolated SLR framework, which is implemented on PyTorch, and trained on an 8GB NVIDIA RTX2080 GPU.

#### Temporal pruning

SL videos in both datasets contain segments in which signers raise and lower their hands at the start and end of each sequence. Since this redundant information dominates the entire frame sequence, which may prevent the model from learning correctly, we drop frames from each sequence temporally by tracking the dominant hand before feeding into our architecture.

#### Spatio-temporal feature extraction

To obtain skeleton joint information, we utilized the publicly available MMPose toolbox.^1^ Skeleton joints are fed into the ST-GCN, which is jointly trained with the sequential architecture. Hand and facial expression representations are extracted using different architectures. To extract hand-shape representations, we use DeepHand (Koller et al., 2016) architecture, pre-trained on over 1 million hand images. We extract facial expression representations using the DAN (Wen et al., 2021) model proposed for the facial expression recognition task and pre-trained on the widely used AffectNet (Mollahosseini et al., 2017) dataset.

Since DeepHand and DAN architecture operate on the region of hand and face, we utilized tracked skeleton joint coordinates to crop around the related region before extracting any features. Then, we extracted *T* × 1024 dimensional hand shape features ${\hat{X}}_{T}^{hand}$ from DeepHand model, and *T* × 512 dimensional facial expression features ${\hat{X}}_{T}^{face}$ from DAN model. After the hand and facial expression features are extracted, they are passed through temporal convolutional layers to adjust their length to the output of the ST-GCN architecture (T^*^), as mentioned in Section 3.1. Additionally, a residual connection from the output ST-GCN architecture to the temporal module is added to avoid overfitting during training. The effects of adding the residual connection can be seen in Table 1.

**Table 1:** Recognition performance on BosphorusSign22k and AUTSL datasets using hybrid ST-GCN and LSTM architecture.

**Architecture**	**Residual**	**BosphorusSign22k**		**AUTSL (Test set)**	
		**Top-1 Acc (%)**	**Top-5 Acc (%)**	**Top-1 Acc (%)**	**Top-5 Acc (%)**
ST-GCN	-	85.86	97.83	86.67	98.16
+ 1 × LSTM	-	84.93	97.83	85.92	97.97
+ 2 × LSTM	-	84.01	97.28	85.82	98.02
+ 3 × LSTM	✓	**88.21**	**98.43**	**87.63**	**98.08**

Details of the ablation study for ST-GCN parameters can be found in the Supplementary material (*t*_*k*_ = 7 is the temporal kernel size and, *t*_*s*_ = {2, 3} is the temporal stride size of the ST-GCN architecture).

#### Temporal modeling and classification

In order to model multiple visual cues at each time step, a single layer of bidirectional LSTM for baseline experiments and MC-LSTM for the proposed framework are employed with the hidden size of 512 with 0.5 dropout for each direction. After MC-LSTM, the output states of each cue are concatenated and averaged over all time steps, which are then used to compute cross-entropy loss.

#### Training and inference

We trained our architecture using Adam (Kingma and Ba, 2014) optimizer with a batch size of 16, the base learning rate of 10^−4^, and weight decay of 10^−4^ for 60 epochs. We perform five warm-up epochs and increase the learning rate by a factor of 10 at epochs 25 and 45 during training. In all of our experiments, we used the same hyperparameters.

The proposed architecture was implemented in PyTorch (Paszke et al., 2019), and pre-trained features were extracted using th Tensorflow implementation of DeepHand (Koller et al., 2016) and PyTorch implementation of DAN (Wen et al., 2021).

### 4.3. ST-GCN with LSTM as a baseline

Sign Languages (SLs) comprise hand articulation, facial expressions and upper body movement. Depending on the resolution, information loss in some of the cues is inevitable when full-image inputs are fed into a CNN-based framework. In addition, skeleton-based inputs are robust to unwanted details in the background using only the information from the body pose. Therefore, we use skeleton-based representations for full body and CNN-based representations for hand and face regions.

Before fusing multiple cues of information with MC-LSTMs, we examine the effects of adding LSTM layers to temporally model ST-GCN representations and train the entire architecture in an end-to-end manner since the proposed framework comprises end-to-end training of an ST-GCN with MC-LSTM. This experiment facilitates the investigation of the multi-cue fusion's contribution. Experiments in Table 1 show that adding LSTM layers right after ST-GCN decreases the recognition performance on both BosphorusSign22k and AUTSL datasets.

In our analysis, we have seen that the model with LSTM layers rapidly overfits during training. To avoid overfitting, we add a residual connection from the ST-GCN output sequence to the output of the LSTM layer before classification, which has increased the classification performance of ST-GCNs by nearly 3% (from 85.86% to 88.21%) on the BosphorusSign22k dataset, and 1% (from 86.67% to 87.63%) on the AUTSL dataset.

### 4.4. Temporally modeling multi-cue information *via* MC-LSTMs

To observe the learning capability of our architecture before multi-cue modeling, we compare the recognition performance when different sets of features are used. Table 2 shows that using hand representations (DeepHand) coupled with LSTM achieved 78.89% recognition performance on the BosphorusSign22k dataset and 60.35% on the AUTSL dataset; adding the residual connection had a minimal effect. Although DeepHand representations do not impact the performance much when used individually, they may still have discriminating information about the hand shape.

**Table 2:** Classification results for multi-cue temporal modeling with MC-LSTM using both manual (ST-GCN and Hand-DeepHand) and non-manual (Face-DAN) representations.

**Multi-cue architecture**	**Sequential Architecture**	**Residual**	**BosphorusSign22k**		**AUTSL (Test Set)**	
			**Top-1 Acc (%)**	**Top-5 Acc (%)**	**Top-1 Acc (%)**	**Top-5 Acc (%)**
ST-GCN	LSTM	✓	88.21	98.43	87.63	98.08
Hand (**L**)		-	78.89	94.74	60.35	85.18
Hand (**L**) + Face	MC-LSTM	-	81.48	96.20	63.85	86.82
Hand (**L**+**R**) + Face			86.59	97.31	71.31	90.38
ST-GCN + Hand (**L**)	MC-LSTM	✓	89.20	96.20	89.68	98.53
ST-GCN + Hand (**L**+**R**)			89.81	98.83	87.68	98.13
ST-GCN + Face			90.08	98.56	87.97	98.40
ST-GCN + Hand (**L**) + Face			**92.58**	99.07	**90.85**	**98.74**
ST-GCN + Hand (**L**+**R**) + Face			91.79	**99.20**	88.92	98.18
ST-GCN + Hand (**L**) + Face	MV-LSTM	✓	91.62	99.09	89.94	98.56

L: Left hand and R: Right hand, the features extracted from pre-trained DeepHand model). MV-LSTM experiment is performed using α = 1 and β = 1 parameters.

Based on this idea, we adaptively fuse hand shape information obtained from DeepHand and facial expression representations from DAN using MC-LSTMs. Our experiments have shown that the fusion of hand shape and face cues has significantly improved the recognition performance (81.48% and 63.85%) compared to using only DeepHand representations (78.89% and 60.35%), empirically showing that there is indeed extra information that can be modeled by using additional cues.

Moreover, we train the ST-GCN architecture with both DeepHand and DAN representations on the multi-cue setup with two or three-channel combinations to investigate the effectiveness of our proposed architecture on the isolated SLR task. As in Table 2, our experiments have shown that utilizing MC-LSTMs for multi-cue fusion has improved our recognition performance (92.58% and 90.85%, BosphorusSign22k and AUTSL datasets, respectively) when all visual cues are combined. We should also note that the ST-GCN module has been jointly trained with MC-LSTM, while representations from other visual cues were only passed through temporal convolutions.

In our experiments, we have also included the non-dominant hand (**R**: right hand) as a separate cue in addition to the dominant hand (**L**: left hand). As seen in Table 2, additional hand representation has improved the architecture's performance significantly (81.48% to 86.59% on BosphorusSign22k and 63.85% to 71.31% on AUTSL datasets) when only DeepHand features are used without the ST-GCN model. The decrease in the recognition performance on both BosphorusSign22k (92.58% to 91.79%) and AUTSL(90.85% to 88.92%) datasets indicates that architecture with ST-GCN can learn most of the information from the dominant hand, already represented by DeepHand features.

### 4.5. Comparison with the state-of-the-art

We compare our best results with the state-of-the-art methods in the literature. Results in Table 3 show that the proposed multi-channel SLR architecture yields competitive recognition performance (92.58%) compared to the state-of-the-art on the BosphorusSign22k (Özdemir et al., 2020) dataset.

**Table 3:** Comparison with the state-of-the-art results on the BosphorusSign22k dataset.

** References**	** Method**	**Channels**				**Top-1 Acc (%)**	**Top-5 Acc (%)**
		**Full**	**Hand**	**Face**	**Pose**		
Özdemir et al. (2020)	MC3 ResNets	✓	-	-	-	78.85	94.76
Kındıroğlu et al. (2019)	Temporal accumulative features (General subset)	-	-	-	✓	81.37	97.47
Gökçe et al. (2020)	MC3 ResNets + ST Sampling	✓	-	-	-	86.91	98.17
Özdemir et al. (2020)	Improved Dense Trajectories	✓	-	-	-	88.53	94.76
Sincan and Keles (2022)	I3D + RGB-MHI Fusion (pretrained on AUTSL)	✓	-	-	-	94.83	-
Gökçe et al. (2020)	MC3 ResNets + ST Sampling + Weighted Score Fusion	✓	✓	✓	-	**94.94**	**99.76**
**Ours**	**ST-GCN + MC-LSTM**	-	✓	✓	✓	**92.58**	**99.07**

Although the single-channel ST-GCN-LSTM-based architecture (88.21%) achieves similar recognition performance with IDT (88.53%) (Özdemir et al., 2016), the IDT approach is highly complex and computationally expensive, making it harder to extract representations and train them for the SLR task. However, our single-cue approach only uses skeleton joint information, which is more accessible and easier to train.

Furthermore, the approach in Gökçe et al. (2020) with the top result (94.94%) in the Table 3 depends upon preprocessing and separate training of multiple 3D CNN architectures for each visual cue, including the entire frame, and fusing their classification scores after they are fully trained while our best result (92.58%) has been achieved by utilizing skeleton joint information and localized representations from pre-trained CNN architectures. Even if a large-scale dataset is available, due to high time and computational complexity, it is not practical to train 3D CNN-based SLR frameworks in an end-to-end manner, which may take days with limited resources. In contrast, our method uses pre-trained models and trains ST-GCN coupled with MC-LSTM, which takes approximately 4 h on a single GPU.

For the AUTSL dataset, our approach yields a promising recognition performance (90.85%) compared to the state-of-the-art methods in the literature. As can be seen in Table 4, researchers have often used the full RGB frame information along with skeleton pose information in their approaches. In addition to the entire frame and skeleton pose, the best performing recognition (98.42%) architecture (Jiang et al., 2021) uses an ensemble approach that utilizes depth and optical flow modalities. Although the architecture in Jiang et al. (2021) has the highest performance, the availability of the features used in the approach may be limited for other resources. Furthermore, ensemble approaches make it difficult to interpret the contributions of different visual SL cues of a multi-cue architecture.

**Table 4:** Comparison with the state-of-the-art results on the test set of the AUTSL dataset.

**References**	** Method**	**Channels**						**Top-1 Acc (%)**	**Top-5 Acc (%)**
		**Full**	**Hand**	**Face**	**Pose**	**Depth**	**Of**		
Sincan and Keles (2020)	2D CNN + BLSTM	✓	-	-	-	-	-	49.22	-
Moryossef et al. (2021)	OpenPose + Holistic	✓	-	-	✓	-	-	81.93	-
De Coster et al. (2021)	VTN-PF	✓	-	-	✓	-	-	92.92	-
Sincan and Keles (2022)	I3D + RGB-MHI Fusion	✓	-	-	-	-	-	93.53	-
Gruber et al. (2021)	I3D + VLE-Transformer	✓	✓	✓	✓	-	-	95.46	-
Vazquez-Enriquez et al. (2021)	MS-G3D + S3D	✓	-	-	✓	-	-	96.15	
Jiang et al. (2021)	SAM-SLR v2	✓	-	-	✓	✓	✓	**98.42**	**-**
**Ours**	**ST-GCN + MC-LSTM**	-	✓	✓	✓	-	-	**90.85**	**98.74**

of: the optical flow modality.

### 4.6. Evaluation of the effect of different cues on sign language attributes

We further analyze the recognition performance on different subsets of sign classes representing linguistic attributes of SLs (as mentioned in Section 4.1); one-handed, two-handed, circular, non-circular, repetitive, non-repetitive, mono-morphemic, and compound signs. In our analysis (shown in Tables 5, 6), we first train the models using the entire dataset, then evaluate only the specific sign class subset and report its recognition accuracy to investigate the effects of different cues on the recognition performance of a sign attribute.

**Table 5:** Top-1 Accuracy (%) of different linguistic attributes on the BosphorusSign22k dataset representing the overall sign recognition performance of each predetermined linguistic sign group.

**Multi-cue architecture**	**Sequential architecture**	**Selected attributes (# classes)**								
		**One handed (234)**	**Two handed (510)**	**Circ. (75)**	**Non Circ. (669)**	**Rep. (457)**	**Non Rep. (287)**	**Mono. (375)**	**Comp. (369)**	**All (744)**
ST-GCN	-	80.76	88.33	91.00	85.38	85.52	86.64	81.29	90.68	85.86
Hand (**L**)	LSTM	77.87	79.70	77.05	77.36	79.29	78.87	74.05	84.25	78.89
ST-GCN		84.55	90.40	91.80	88.20	88.39	88.84	86.24	90.92	88.21
Hand (**L**) + Face	MC-LSTM	81.57	81.53	81.85	81.50	82.15	80.57	77.59	85.52	81.48
Hand (**L**+**R**) + Face		83.70	88.11	88.90	86.48	88.09	84.55	83.10	90.44	86.59
ST-GCN + Hand (**L**)	MC-LSTM	84.83	91.17	89.82	89.11	89.19	89.16	85.01	93.41	89.20
ST-GCN + Hand (**L**+**R**)		85.23	91.95	91.98	89.60	90.33	89.06	86.77	92.96	89.81
ST-GCN + Face		88.61	91.69	89.98	90.81	90.63	90.86	88.37	93.09	90.08
ST-GCN + Hand (**L**) + Face		**90.00**	**93.79**	**93.33**	**92.52**	**92.84**	**92.21**	**90.76**	**94.48**	**92.58**
ST-GCN + Hand (**L**+**R**) + Face		89.58	92.68	90.84	91.80	92.13	91.02	89.90	93.53	91.79

Numbers below each attribute show the number of classes belonging to that attribute.

**Table 6:** Top-1 Accuracy (%) of different linguistic attributes on the AUTSL dataset representing the overall sign recognition performance of each predetermined linguistic sign group.

** Multi-cue architecture**	**Sequential architecture**	**Selected attributes (# classes)**								
		**One handed (105)**	**Two handed (121)**	**Circ. (7)**	**Non Circ. (219)**	**Rep. (87)**	**Non Rep. (139)**	**Mono. (194)**	**Comp. ( 32)**	**All (226)**
ST-GCN	-	84.93	87.89	90.55	86.38	86.62	86.45	85.87	90.39	86.67
Hand (**L**)	LSTM	59.29	61.78	41.23	60.76	61.10	59.57	59.45	64.44	60.35
ST-GCN		85.16	89.71	87.03	87.61	87.88	87.42	86.87	92.00	87.63
Hand (**L**) + Face	MC-LSTM	63.67	63.63	62.55	63.68	62.16	64.58	62.91	68.15	63.85
Hand (**L**+**R**) + Face		65.47	76.14	81.83	70.84	71.98	70.68	69.78	79.66	71.31
ST-GCN + Hand (**L**)	MC-LSTM	88.33	90.75	90.39	89.60	89.58	89.65	88.97	93.60	89.68
ST-GCN + Hand (**L**+**R**)		85.24	89.97	85.98	87.83	87.80	87.76	86.80	93.68	87.68
ST-GCN + Face		85.89	89.73	**90.60**	87.86	88.16	87.81	87.31	91.79	87.97
ST-GCN + Hand (**L**) + Face		**89.46**	**92.01**	89.65	**90.86**	**91.54**	**90.38**	**90.30**	**94.03**	**90.85**
ST-GCN + Hand (**L**+**R**) + Face		87.02	91.04	**90.60**	89.13	89.94	88.69	88.53	93.06	88.92

Numbers below each attribute show the number of classes belonging to that attribute.

Results in both BosphorusSign22k and AUTSL datasets show that multi-channel hybrid architecture with ST-GCNs and MC-LSTMs performs well on all sign attributes subsets, except on the circular signs of the AUTSL dataset, which is related to the number of classes in the subset. Furthermore, our analysis demonstrates that using an architecture that utilizes skeleton joint information with ST-GCNs outperforms feature-based sequential architectures in all cases. Especially for the compound signs, our approach performs the best among all other attributes. As compound signs are composed of multiple hand shapes and high movement compared with the other signs, we believe ST-GCNs can model such complex characteristics better than feature-based approaches.

Furthermore, our analysis shows that the recognition performance of the compound signs is also higher when the multi-cue ST-GCN-MC-LSTM architecture is used where skeleton joint and feature-based information are used together. Since compound signs are often longer than other signs and have complex spatio-temporal characteristics, we believe that employing a multi-cue sequential architecture such as MC-LSTMs has further improved recognition performance.

## 5. Conclusion

This study aims to improve the performance of isolated SLR by exploiting multi-articulatory spatio-temporal information from both manual (hand shapes and gestures) and non-manual (facial expression) features. In addition to the performance improvement, we intend to shed light upon the individual contribution of different cues on the recognition performance of the sign glosses. For this purpose, we propose a skeleton-based SLR architecture employing ST-GCNs and MC-LSTMs, which learns to fuse the pose information and visual representations extracted from pre-trained DeepHand and DAN architectures. While the features learned by ST-GCN and extracted from pre-trained CNNs provide spatial and spatio-temporal representations, the MC-LSTM carries out the temporal modeling of multiple visual cues by adaptively fusing them at each time step. The proposed temporal pooling approach for the ST-GCN module makes it suitable to train with a sequential model, unlike the standard ST-GCN architecture. Furthermore, we designed MC-LSTMs to learn the cross-cue and cue-specific interaction matrices from data as opposed to using pre-determined values.

We evaluate our approach on the publicly available BosphorusSign22k and AUTSL datasets and obtain comparable recognition performance with the skeleton-based state-of-the-art. Moreover, we extensively analyze different subsets representing linguistic sign attributes, revealing that our multi-cue architecture can exploit complex characteristics of SLs. Our experiments provide empirical evidence that the proposed ST-GCN and MC-LSTM-based framework can model the interactions between multiple visual SL cues without using the information from the entire frame.

## Data availability statement

Publicly available datasets were analyzed in this study. Datasets can be found at: https://ogulcanozdemir.github.io/bosphorussign22k/; https://chalearnlap.cvc.uab.cat/dataset/40/description/.

## Ethics statement

Written informed consent was obtained from the individual(s) for the publication of any potentially identifiable images or data included in this article.

## Author contributions

OÖ implemented and performed the experiments. All authors contributed to the research design, reviewed the results, and approved the final version of the manuscript.

## Footnotes

^1^ https://github.com/open-mmlab/mmpose
