# Supplementary material for: Multi-cue temporal modeling for skeleton-based sign language recognition
Source: Front Neurosci. 2023 Apr 5;17:1148191. doi: 10.3389/fnins.2023.1148191 (PMC10113557; doi:10.3389/fnins.2023.1148191)
Supplement: Supplementary file 1 [file Data_Sheet_1.PDF]

Multi-Cue Temporal Modeling for Skeleton-based Sign Language Recognition Supplementary Material

This supplementary material provides the ablation study, which is not shown in the manuscript. In the study, we analyze adjusting the Spatial-Temporal Graph Convolutional Network (ST-GCN) hyperparameters, temporal kernel size t_k, and temporal stride t_s for our Sign Language Recognition (SLR) framework. We first present the ablation study on temporal kernel size t_k (Section 1), and then we provide our analysis for the temporal stride parameter (Section 2).

1 ABLATION ON TEMPORAL KERNEL SIZE

We start our analysis on the effect of adjusting the temporal kernel size of the baseline ST-GCN architecture to adapt it to our multi-cue sequential architecture on the BosphorusSign22k dataset. We first fix the temporal stride of ST-GCNs to $\{2, 2\}$, where the number of channels in the feature maps is increased, and train the baseline architecture for varying temporal kernel sizes t_k to investigate the changes in the temporal domain.

Experiments in Table S1 have shown that slight changes in the temporal kernel have minimal effects on recognition performance. In contrast, significant changes to the baseline kernel size of 9 (Yan et al. (2018)) reduce performance. Although the architecture yields similar performance at 7 (85.68%) and 9 (85.15%) temporal kernel sizes, we chose $t_k = 7$ for the rest of our experiments.

Table S1. Effects of different temporal kernel sizes (t_k) on the recognition performance of the baseline ST-GCN architecture with fixed temporal stride $t_s = \{2, 2\}$ on the BosphorusSign22k dataset. (* denotes the temporal kernel size from the baseline ST-GCN architecture.)

Temporal kernel size (t_k)	Top-1 Acc (%)	Top-5 Acc (%)
3	74.11	94.78
5	82.87	97.07
7	**85.68**	**97.69**
9*	85.15	97.48
11	84.98	97.49
13	83.72	96.67

2 ABLATION ON TEMPORAL STRIDE

We investigate changing the temporal stride parameter, resulting in different output sequence lengths. Although temporal strides of $\{2, 3\}$ and $\{3, 2\}$ result in the same output sequence lengths, they did not give the same recognition performance (85.86% and 85.81%) (shown in Table S2). This may be caused by collapsing important temporal information too early. For the rest of our experiments, we fix the temporal stride of the same ST-GCN blocks to $\{2, 3\}$.

REFERENCES

Yan, S., Xiong, Y., and Lin, D. (2018). Spatial temporal graph convolutional networks for skeleton-based action recognition. In *Thirty-second AAAI conference on artificial intelligence*

Table S2. Effects of different temporal strides (t_s) on the recognition performance of the baseline ST-GCN architecture with fixed temporal kernel size $t_k = 7$ on the BosphorusSign22k dataset.

Temporal stride (t_s)	Top-1 Acc (%)	Top-5 Acc (%)
{1, 1}	82.49	96.68
{1, 2}	83.06	96.79
{1, 3}	84.05	97.26
{2, 1}	84.78	97.91
{2, 2}	85.68	97.69
**{2, 3}**	**85.86**	**97.83**
{3, 1}	85.48	97.67
{3, 2}	85.81	97.69
{3, 3}	85.30	97.66
